# Integrating prior knowledge inference with computational multi-omics analysis to reveal host antiviral networks of natural compounds against influenza A virus

**DOI:** 10.3389/fcimb.2026.1771638

**Published:** 2026-03-02

**Authors:** Xu Chen, Jing Ma, Yuan Wang, Ying Dang, Yu-Qi Jiao, Ri Hai, Hong-Lu Ma, Jia-Mei Zhang, Xiao-He Li, Jian-Ping Shi

**Affiliations:** 1 College of Traditional Chinese Medicine, Inner Mongolia Medical University, Hohhot, Inner Mongolia, China; 2 Department of Ophthalmology, Peking Union Medical College Hospital, Beijing, China; 3 College of Basic Medical Sciences, Inner Mongolia Medical University, Hohhot, Inner Mongolia, China

**Keywords:** aloe emodin, andrographolide, cryptotanshinone, emodin, influenza A virus (IAV), multi-omics analysis, natural compounds

## Abstract

**Introduction:**

Influenza A virus (IAV) remains one of the major global health threats, and a rapid emerging resistance to direct acting antivirals underscores the need for host directed therapies.

**Methods:**

Here, we integrated prior knowledge inference with a multilevel phenotypic screening and computational multi-omics analysis to identify natural candidate chemical compounds from Traditional Chinese Medicine (TCM)–derived libraries. Twenty compounds enriched for links to antiviral signaling were tested via a GFP-IAV reporter assay, followed by experimental validation using replication kinetics of wild type IAV in MDCK and A549 cells, cytotoxicity measurements, and a viral polymerase minigenome assay.

**Results:**

Four structurally distinct compounds, Aloe emodin, Cryptotanshinone, Emodin, and Andrographolide, consistently inhibited IAV replication with low cytotoxicity. The polymerase assay results indicated no substantial direct inhibition of viral polymerase, except for modest effects of Aloe emodin at high concentration. Transcriptomic and proteomic profiling of compound-treated and virus-infected A549 cells showed that all four compounds reprogrammed host antiviral and inflammatory networks, including innate immune and stress response pathways, and virus-host interaction modules.

**Discussion:**

These findings nominate the four natural chemical compounds as promising antiviral scaffolds against IAV.

## 1 Introduction

Influenza A virus (IAV) remains one of the formidable and persistent global health threats, posing a potential risk of devastating pandemics (Singh et al., 2025). The World Health Organization (WHO) estimates that seasonal influenza results in approximately one billion infections, 3–5 million cases of severe illness, and 290,000 to 650,000 respiratory deaths annually worldwide (WHO, 2025). The influenza pandemic of H1N1 serotype (Neumann et al., 2009) or seasonal endemic of H3N2 serotype (Potter et al., 2019) underscores the unpredictability of IAV and the constant potential risk of emerging devastating influenza strain via reassorting with H5N1 (Nguyen et al., 2025; Sun et al., 2014; Wei et al., 2025), H5N5 (Taylor, 2025), H7N9 (Deng et al., 2017; Liu et al., 2014; Liu et al., 2014) and other avian IAV serotypes. This evolutionary capacity of IAVs allows the virus to evade pre-existing population immunity, making it a recurrent challenge for public health systems (Lin et al., 2025; Maurer et al., 2025). The clinical and economic burdens associated with these outbreaks, including healthcare costs and lost productivity, further amplify the necessity for continuous and vigilant management of influenza.

The clinical management of influenza infection relies on a limited arsenal of antiviral drugs, which have evolved over time in response to emerging resistance. The first class of antivirals, the M2 ion channel inhibitors (amantadine and rimantadine) targeted the M2 protein of IAV (Kumar and Sakharam, 2024) to inhibit the uncoating of the viral genome within the host cell, and thus halting replication (Kumar and Sakharam, 2024; Mtambo et al., 2021). However, their utility was drastically curtailed by the rapid and global emergence of resistant strains, particularly those with an S31N mutation in the M2 gene, which is now pervasive in circulating IAV (Deng et al., 2024; Wang et al., 2011). Compounding the problem of resistance were concerns over neurotoxic side effects associated with amantadine (Yang et al., 2025). Consequently, these drugs are no longer recommended for the treatment of influenza, marking a significant early setback in antiviral therapy. The limitations of the adamantanes led to the development and adoption of neuraminidase inhibitors (NAIs), which became the cornerstone of influenza treatment for decades, such as oseltamivir (Tamiflu) and zanamivir (Relenza) (Doshi et al., 2016; Jackson et al., 2011). By inhibiting neuraminidase, NAIs prevent the progeny viruses from being released from infected cells (Chauhan and Gordon, 2022), thereby containing the infection within the respiratory tract. Despite its proven efficacy in reducing the duration of symptoms and mitigating complications when administered early, NAI is worrisome because of the easy emergence of NAI-resistant strains, due to the IAV’s high mutation rate and error-prone RNA-dependent RNA polymerase (RdRP). Surveillance studies have documented mutations in the neuraminidase gene (e.g., H275Y) that confer reduced susceptibility to oseltamivir, highlighting a vulnerability in this therapeutic mainstay (Ison, 2011). The more recently approved baloxavir marboxil, a cap-dependent endonuclease inhibitor, marked a significant advance by targeting the viral polymerase complex (Dufrasne, 2021). While highly effective, evidence suggests that IAVs can also develop reduced susceptibility to baloxavir, highlighting an ongoing and critical challenge: the relentless evolution of the virus demands a continuous pipeline of novel antiviral agents with distinct mechanisms of action to stay ahead of resistance.

This pressing need has catalyzed a paradigm shift in antiviral drug discovery toward Host-Directed Therapies (HDTs). Unlike conventional antivirals that target viral components, which are mutable and can lead to resistance, HDTs aim to target host cellular factors that are hijacked by the virus for its replication. Since host factors are genetically more stable, this strategy offers the potential for broad-spectrum efficacy against diverse IAV strains and a higher genetic barrier to resistance (Gao et al., 2022; Haas et al., 2023). The development of HDTs represents a crucial frontier in our defensive strategy against influenza infection. In the quest for such host-directed agents, natural chemical compounds derived from Traditional Chinese Medicine (TCM) systems present an invaluable and rich resource. These compounds have evolved in conjunction with biological systems and often exhibit multi-targeted activities, making them ideal starting points for discovering HDTs. For instance, TCM, steeped in centuries of empirical knowledge and holistic practice, has been used to treat infectious diseases, including those involving fever and respiratory symptoms, suggesting a repository of potential antiviral agents (Han et al., 2023; Shang et al., 2024). Notably, specific natural compounds like Kaempferide (a flavonoid from Scutellaria baicalensis and Andrographis paniculata) have been reported to possess antiviral activities against IAV (Nie et al., 2024), among other pharmacological effects (Song et al., 2024). Similarly, other compounds such as silymarin have also been investigated for their biological activities (Song and Choi, 2011). This historical use, combined with modern pharmacological findings, provides a strong rationale for systematically exploring traditional compound libraries to identify novel anti-IAV leads.

To this end, we designed a multi-level screening strategy aimed at identifying compounds with anti-IAV activity from a library of natural chemical compounds derived from traditional medicine. The strategy first anchored to the representative traditional medical literature—the Pharmacopoeia of the People’s Republic of China. Through literature mining, compound database searches, virtual screening based on structural similarity, and comparative analysis with known natural chemical compounds exhibiting anti-influenza activity, we screened 50 representative compounds with potential antiviral activity from hundreds of TCMs listed in the Pharmacopoeia. Subsequently, we conducted manual precision screening by integrating prior knowledge, focusing on compounds with activity related to “antiviral signaling pathways.” Following this rigorous stepwise screening process, 20 compounds with potential IAV activity were ultimately identified as candidate compounds for subsequent experimental validation. Following preliminary screening of 20 compounds, a further screening was conducted using a GFP-based reporter virus system to identify candidate compounds with potent antiviral activity. The most promising candidates were validated through viral replication assays. To further elucidate their mechanisms of action, we investigated the effects of these compounds on viral polymerase activity and evaluated their impact on host cell antiviral signaling pathways via omics analysis. Through this comprehensive workflow, we successfully screened out four natural compounds with significant anti-IAV activity, each exerting effects through distinct host-directed mechanisms, providing novel intervention pathways for IAV treatment.

## 2 Materials and methods

### 2.1 Reagents, cells, and viruses

MDCK (Madin-Darby canine kidney) cells (ATCC, Manassas, VA, USA, CCL-34), and HEK293T (Human embryonic kidney) cells (ATCC, CRL-3216) were cultured at 37°C, 5% CO_2_ in Dulbecco’s modified Eagle’s medium (DMEM, Thermo Fisher Scientific, Waltham, MA, USA) supplemented with 10% heat-inactivated fetal bovine serum (Gibco) and 1% PSG (100 U/mL penicillin, 100 μg/mL streptomycin). A549 (Human lung epithelial carcinoma) cells (ATCC, CCL-185) were cultured at 37°C, 5% CO_2_ in F-12K Nutrient Mixture (F-12K, Thermo Fisher Scientific) supplemented with 10% heat-inactivated fetal bovine serum (Gibco) and 1% PSG (100 U/mL penicillin, 100 μg/mL streptomycin). H1N1 subtype IAV A/California/07/2009 (CA07) were propagated in MDCK cells. The viruses carrying the gene of green fluorescent protein (GFP) used in this experiment were provided by the Key Laboratory of Pathogens and Biosecurity at the Academy of Military Medical Sciences. All the Chinese herbal compounds needed for the experiments were purchased from MCE.

### 2.2 Fast and high-throughput screening for anti-IAV compounds with GFP-IAV reporter

Well-grown MDCK cells were inoculated at 250 μL of medium per well into a 48-well plate. After ensuring uniform cell distribution, the plate was gently transferred to a 37°C, 5% CO_2_ incubator for continued cultivation. When the cell confluence reached over 80%, the medium in each well was aspirated and replaced with virus isolation serum-free medium. A 200 μL mixture of GFP-labeled influenza virus (Sun et al., 2014) was added and incubated for 1 h at an multiplicity of infection (MOI) of 0.001. The plate was shaken every 15 minutes to prevent cell clumping. Compounds were diluted to 50 μM and prepared as 250 μL solutions in the plate, with one duplicate well for each compound. The plates were then cultured again in the 37°C, 5% CO_2_ incubator. At 12, 18, and 24 h post-infection, cell status and GFP fluorescence intensity were observed using Gen5 fluorescence microscopy. The viral infection-induced GFP expression and its changes under drug treatment were analyzed. Fluorescence intensity measurements were employed to evaluate viral infectivity and drug efficacy against the infection. Preliminary screening identified compounds with significant antiviral activity.

### 2.3 Quantitative real-time PCR

Total RNA was extracted from virus culture supernatant using a PureLink RNA Mini Kit (Thermo Fisher Scientific). qRT-PCR was performed using a One Step TB Green PrimeScript PLUS RT-PCR Kit (TaKaRa, Shiga Prefecture, Kusatsu City, Japan) with a Light Cycler 480 II (Roche, Basel, Switzerland). Virus titer was determined by qRT-PCR and converted to plaque-forming units (PFU) numbers by standard curve.

### 2.4 Viral replication dynamics experiment

MDCK cells were cultured at 500 μL of medium per well in a 24-well plate. After achieving uniform cell coverage, the plates were transferred to a 37°C, 5% CO_2_ incubator for continued culture. When cell confluence exceeded 80%, the original medium was aspirated and replaced with serum-free viral isolation medium. 200 μL of wild-type CA07 virus was added and incubated at an MOI of 0.001 for 1 h. The plates were shaken every 15 minutes to prevent clumping. The compounds were diluted to 50 μM and prepared as 1800 μL of solutions, with three replicate wells per compound. The plates were then returned to the incubator for further culture. At 12, 24, 48, and 72 h post-infection, cell viability was assessed under a microscope, and 230 μL of supernatant was collected for nucleic acid extraction and qRT-PCR to evaluate antiviral efficacy. Viral replication kinetics experiments confirmed that the four compounds exhibited antiviral activity. A549 cells were cultured using F-12K medium at an MOI of 0.01, following the same protocol.

### 2.5 Cell viability assay

MDCK and A549 cells were seeded in 96-well plates and incubated at 37°C with 5% CO_2_ for over 12 h. After confirming cell adhesion, the plates were washed three times with PBS, followed by the addition of 100 μL of virus-isolated serum-free medium containing the compound at 200 μM starting concentration. Six duplicate wells were prepared for each concentration, with one column containing only medium as the blank control and another containing DMSO as the negative control. The plates were incubated at 37°C for 24 h, then allowed to equilibrate at room temperature for 30 minutes. Two hours prior, the CellTiter-Glo Reagent (Promega) was thawed, and 100 μL of substrate-buffer mixture was added per well. After mixing, the plates were incubated on a shaker for 2 minutes to lyse cells. The plates were then incubated at room temperature for 10 minutes to stabilize luminescence signals, followed by automated detection. Cell survival rate (%) = (the absorbance of drug-treated group/the absorbance of blank control group) × 100%.

### 2.6 Dual luciferase assay for detection of compound expression levels

293T cells were prepared for 24 h in advance. Plate them in 48-well plates and incubate overnight at 37°C with 5% CO_2_. When cells reach 80% confluence, they are then transfected with Lipofectamine 3000 transfection kit to introduce IAV *PB1, PB2, PA, NP*, pHH21-Fluc, and pRL-TK plasmids. After a 5-minute incubation separately, liposomes and genetic material were mixed and incubated for more than 15 minutes, were supplemented with 25 μL of DNA-liposome complex per well, and then were incubated at 37°C with 5% CO_2_ for 6 h. Finally, the cell supernatant was removed and the cells were updated with compound-containing medium. Six gradient concentrations (50 μmol/mL total) were prepared using three sub-wells per gradient, with Baloxavir marboxil as positive control, and with DMSO and blank as negative controls. Cells for all groups were maintained at 37°C with 5% CO_2_ for 24 h, and then both Firefly luciferase (Fluc) and Renilla reniformis (Rluc) were detected using Promega Dual-Luciferase Reporter Assay System kit, and the Fluc/Rluc was calculated.

### 2.7 Transcriptome and proteome sequencing analysis

The A549 cells were inoculated into a 6-well plate at a density of 2 mL of medium per well. After the cells were evenly spread, they were transferred to a 37°C, 5% CO_2_ incubator for further culture. When the cell density reached 80-90%, the cells were washed three times with PBS and replaced with serum-free medium. A 500 μL suspension of wild-type CA07 virus was added, and the cells were incubated at an infection multiplicity (MOI = 0.1) for 1 h. The cells were shaken every 15 minutes to prevent clumping. The compound was diluted to the corresponding concentration and prepared into a 7 mL solution, with concentrations of Andrographolide (50 μM), Cryptotanshinone (6.25 μM), Aloe emodin (25 μM), Emodin (25 μM), and DMSO as the negative control. Three replicate wells were set for each compound, and the culture plates were returned to the incubator for continued cultivation. After an incubation for 24 h, the cell status was observed, and the medium was aspirated. TRIzol^®^ reagent (Thermo Fisher Scientific) was used to extract total RNA for transcriptome analysis. The medium was removed, and the cells were washed with PBS before being digested with trypsin to collect proteins for proteome sequencing analysis. For all the above compounds, a control group without wild-type CA07 virus was established, using medium containing 10% FBS, with other procedures remaining unchanged. The transcriptome and proteome sequencing analyses were performed using GENE DENOVO software (China Guangzhou). Functional annotation and enrichment analysis were performed using the Kyoto Encyclopedia of Genes and Genomes (KEGG) database’s biological processes (BP), cellular components (CC), and molecular functions (MF) classifications.

### 2.8 Statistical analysis of biological results

The GraphPad Prism 9.0 software was used to conduct all statistical analysis. Data were presented as means ± SD of at least three independent experiments. Differences between the two groups were evaluated using Student’s t-test, and differences between three or more groups were evaluated using analysis of variance (ANOVA). statistical significance was set at *p* < 0.05.

## 3 Results

### 3.1 Rapid screening of antiviral drugs using GFP-IAV

**Figure 1:**
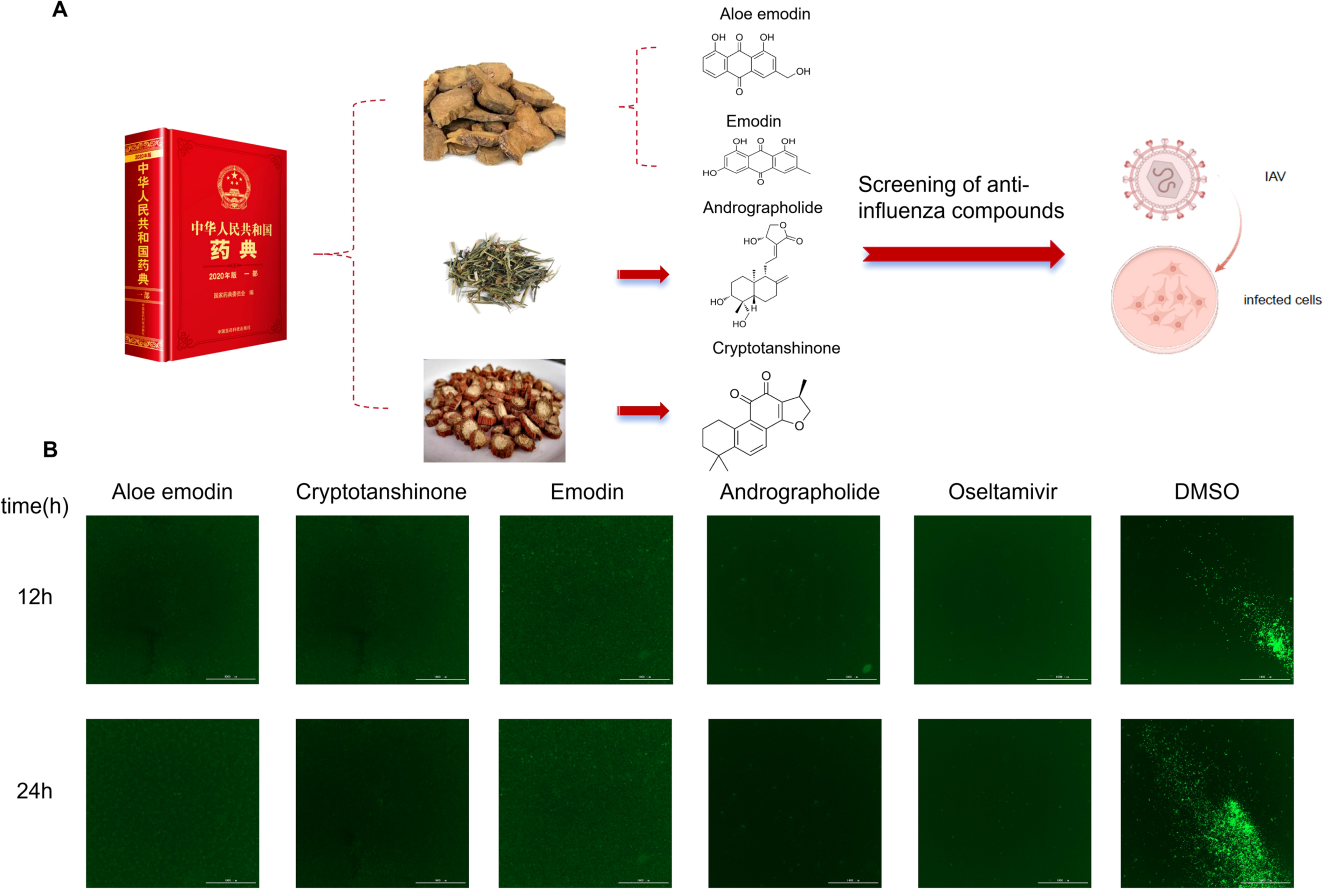
Four compounds were screened from the natural chemical compounds library derived from traditional medicine. **(A)** Workflow for identifying anti-IAV compounds from natural chemical compound libraries of traditional medicinal sources and schematic chemical structures of four compounds. **(B)** MDCK cells infected with GFP-IAV were treated with candidate antiviral drugs. If the drug exhibits antiviral activity against IAV, GFP expression decreases; conversely, higher GFP levels indicate poor antiviral efficacy.

Natural compound libraries often contain hundreds of natural compounds with diverse functions. To improve screening efficiency and narrow the screening scope, we adopted a method combining literature mining, compound database screening, and virtual screening based on structural similarity, supplemented by prior knowledge for manual precision screening. First, we selected the representative traditional medical literature, the Pharmacopoeia of the People’s Republic of China, as the target text. Through literature mining, compound database screening, virtual screening based on structural similarity, and comparative analysis with known natural chemical compounds exhibiting antiviral activity against influenza viruses, we identified 50 representative compounds with potential antiviral activity from hundreds of TCM materials listed in the Pharmacopoeia. Subsequently, we integrated prior knowledge for manual precision screening and referenced the antiviral activity annotations of compounds on the MCE website to clarify the screening direction. The primary screening targets were compounds with activity related to “antiviral signaling pathways,” including those capable of modulating the interferon (IFN) pathway, NF-κB signaling pathway, JAK-STAT signaling pathway, MAPK signaling pathway, or natural chemical compounds reported to exhibit antiviral activity against other RNA viruses (e.g., RSV, HCV, SARS-CoV-2, EV71, etc.) (**Figure 1A**). After this rigorous stepwise screening process, we ultimately identified 20 compounds with potential IAV activity as candidate compounds (**Supplementary Table 1**). To evaluate the antiviral activity of selected compounds against IAV, MDCK cells were infected with GFP-IAV and added 50 μM of the natural compound to the supernatant, using DMSO as the negative control and oseltamivir as the positive control. In this screening system, GFP fluorescence intensity directly reflects viral replication within cells, and stronger GFP fluorescence intensity indicates higher viral replication efficiency (Nie et al., 2024). The detection results 24 h after viral infection showed that compared with the DMSO negative control group, the GFP fluorescence intensity in the Aloe emodin, Cryptotanshinone, Emodin, and Andrographolide-treated groups was significantly reduced (**Figure 1B**), indicating that these four compounds could effectively inhibit the replication of IAV in MDCK cells, preliminarily confirming their anti-IAV activity. The chemical structures of the four aforementioned active compounds are illustrated in **Figure 1A**. In summary, through a multi-tiered screening system of “preliminary virtual screening-manual precision screening-GFP reporter virus validation”, we have preliminarily identified four natural compounds with confirmed anti-IAV activity: Aloe emodin, Cryptotanshinone, emodin, and Andrographolide. These compounds provide important candidate molecules for further investigation of their antiviral mechanisms and the development of novel anti-IAV drugs.

### 3.2 Verification of antiviral activity against IAV by viral replication kinetics

**Figure 2:**
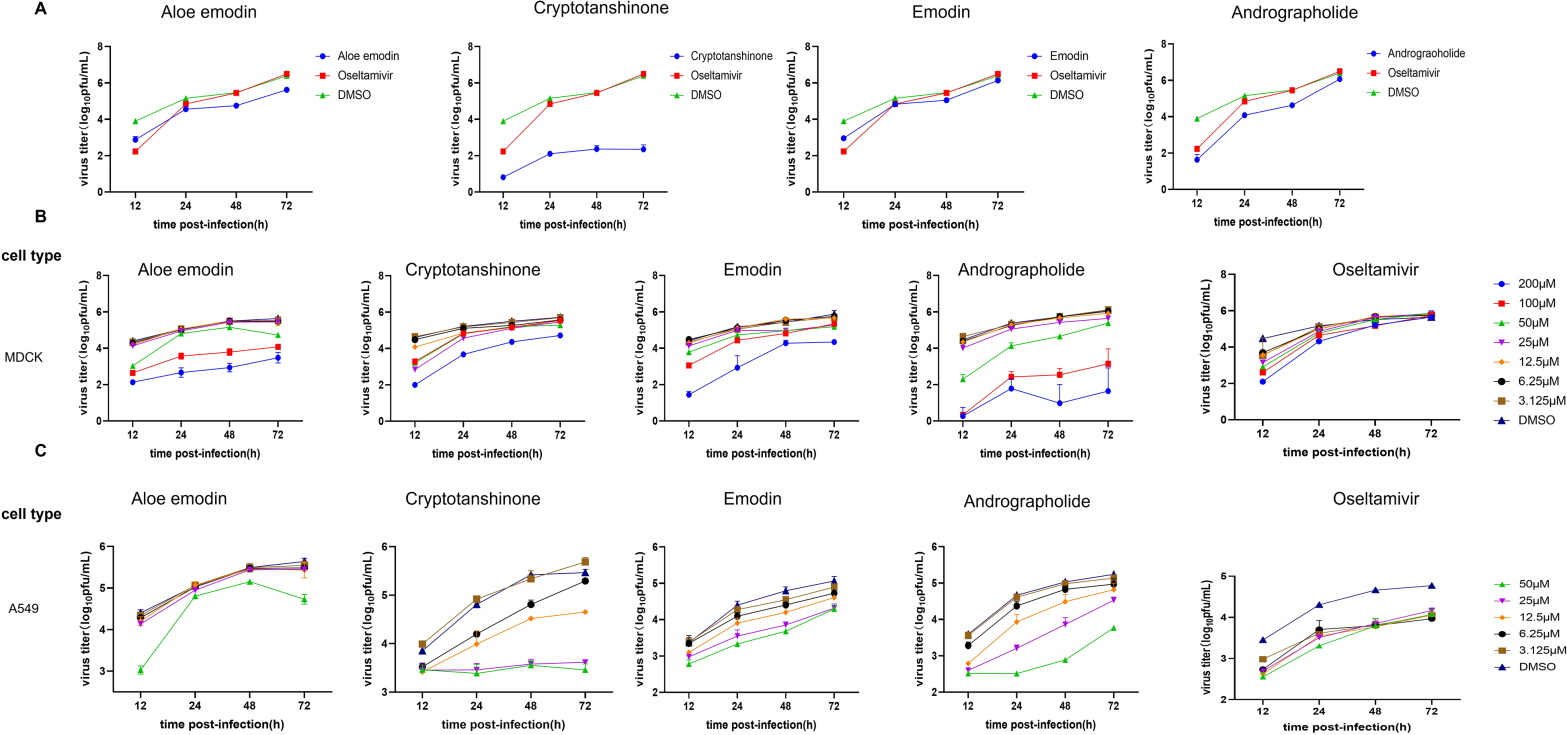
The four compounds showed significant inhibitory effects on wild type IAV. **(A)** Viral replication kinetics experiments were conducted to further validate antiviral activity. Oseltamivir served as the positive control, and DMSO (50 mM) as the negative control. Viral titers were measured at four time points via qRT-PCR, and the data were converted to PFU number using a standard curve to analyze the viral growth curve. **(B)** Viral replication kinetics experiments were performed to validate the antiviral activity of the four compounds on MDCK cells (MOI = 0.001) and determine their optimal concentrations. **(C)** Viral replication kinetics experiments were conducted to validate the antiviral activity of the four compounds on A549 cells (MOI = 0.01) and determine their optimal concentrations.

To further validate antiviral efficacy of these four compounds against IAV, MDCK cells were subsequently infected with wild-type CA07 virus and added natural compounds Aloe emodin, Cryptotanshinone, emodin, and Andrographolide to the supernatant. Oseltamivir was used as the positive control and DMSO as the negative control. Virus titers were measured at different time points (12, 24, 48, or 72 h post-infection) using qRT-PCR. Results showed that the viral replication efficiency in cells treated with the four compounds was significantly lower than that in the DMSO group (**Figure 2A**). To further confirm their antiviral activity in different cell lines and determine optimal concentrations, experiments were conducted using MDCK cells (MOI = 0.001) and A549 cells (MOI = 0.01) infected with wild-type CA07 virus. After 1-hour adsorption, the four compounds were diluted in a 2-fold concentration gradient (200, 100, 50, 25, 12.5, 6.25 and 3.125 μM) and then incubated with infected cells. Oseltamivir served as the positive control, while DMSO as the negative control. qRT-PCR was employed to measure viral titters at each of the four time points (12, 24, 48, or 72 h post-infection). qRT-PCR results showed that the compounds significantly reduced IAV replication in both MDCK and A549 cells compared with the DMSO control (**Figures 2B, C**). At higher concentrations (100 and 200 µM), however, potential effects on cell viability were observed and require further validation. The above results indicated that these four compounds have an inhibitory effect on IAV proliferation, but the specific mechanisms involved require further investigation.

### 3.3 Effects of compounds on host cell viability and IAV polymerase activity

**Figure 3:**
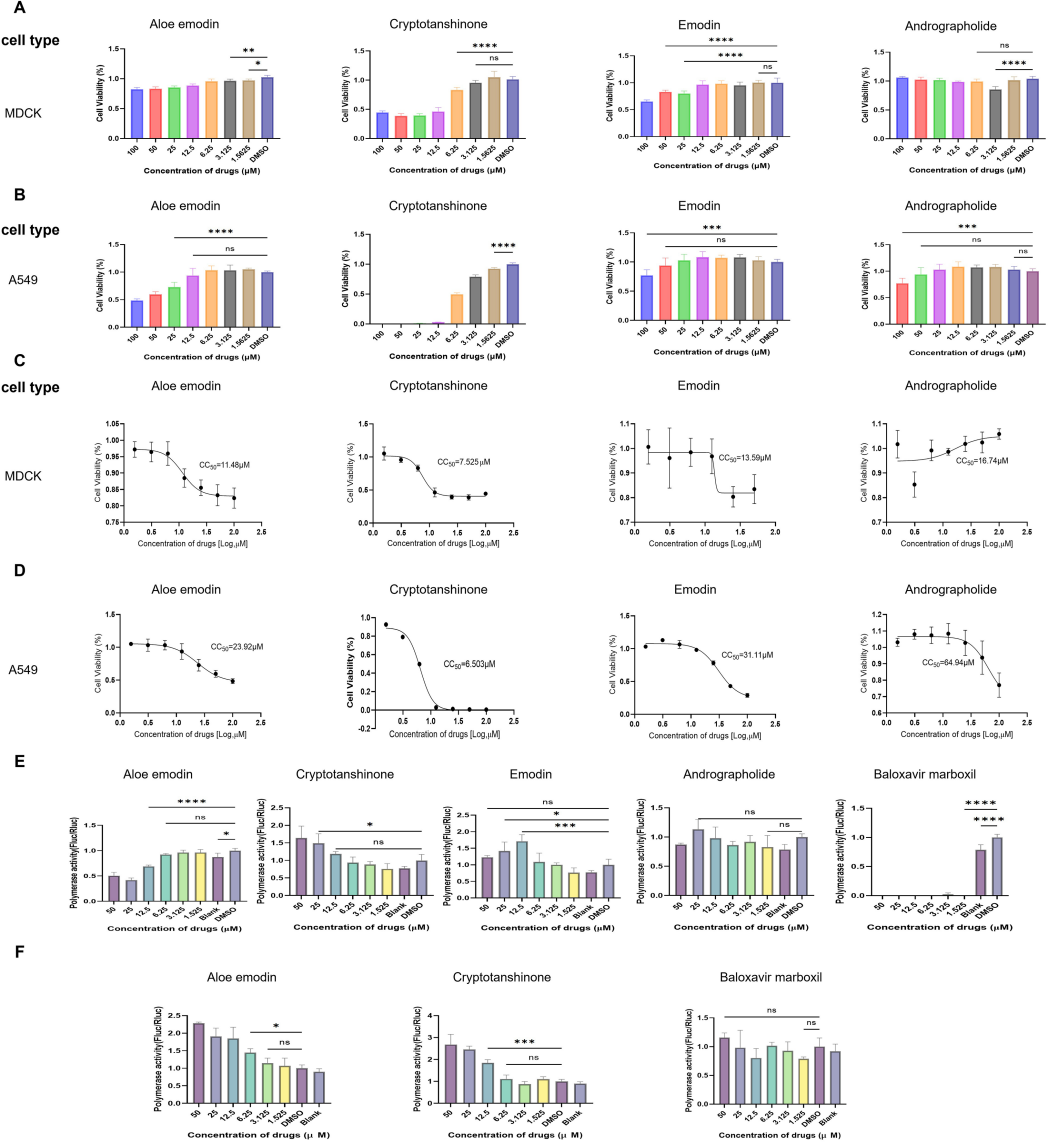
Cell Activity and Polymerase Activity of Four Compounds **(A)** Cell viability of compounds at different concentrations (initial concentration: 100 μM, 2-fold dilution, 6 replicates per concentration) on MDCK cells. **(B)** Cell viability of compounds at different concentrations (initial concentration: 100 μM, 2-fold dilution, 6 replicates per concentration) on A549 cells. **(C, D)** Calculation of median cytotoxicity (CC50) of the four compounds in MDCK and A549 cells, respectively. **(E)** The 293T cells were transfected with plasmids PB1, PB2, PA, NP, pHH21-Fluc, and pRL-TK. The effects of the four compounds on polymerase activity were detected. **(F)** The effects of Aloe emodin and Cryptotanshinone on luciferase activity were investigated in 293T cells transfected with plasmids pcDNA3.1-Fluc and pRL-TK. All statistical significance was determined by two-way ANOVA. All statistical results are labeled as *p < 0.05, **p < 0.01, ***p < 0.001, ****p < 0.0001.

To evaluate the cytotoxicity of these compounds, researchers treated MDCK and A549 cells with a starting concentration of 100 μM, which was then diluted twofold. Each experimental group included a blank control and a DMSO-negative control. Cell viability was assessed after 24 h (**Figures 3A, B**), and the median cell lethal concentration (CC50) for the four compounds was calculated (**Figures 3C, D**). The results showed that in MDCK cells, the CC50 values for Aloe emodin, Cryptotanshinone, Emodin, and Andrographolide were 11.48 μM, 7.525 μM, 13.59 μM, and 16.74 μM, respectively (**Figure 3C**). In A549 cells, the CC50 values were 23.92 μM, 6.503 μM, 31.11 μM, and 64.94 μM, respectively (**Figure 3D**). These compounds exhibited cell-type-dependent toxicity. Further analysis revealed that Cryptotanshinone demonstrated the strongest cytotoxicity in both cell types, with CC50 values below 8 μM. Notably, its toxicity in A549 cells (6.503 μM) was slightly higher than in MDCK cells (7.525 μM), making it the only component among the four compounds that showed superior toxicity in tumor cells (A549) compared to normal epithelial cells (MDCK). In contrast, Andrographolide exhibited the weakest cytotoxicity, with a CC50 value as high as 64.94 μM in A549 cells, which was 3.88 times higher than its CC50 value in MDCK cells, demonstrating the most significant cell type-dependent difference. Aloe emodin and Emodin shared similar toxicological profiles, both showing stronger toxicity to normal MDCK cells.

To investigate whether the antiviral effects of compounds were related to inhibition of IAV polymerase activity, the viral polymerase activity was detected by a dual-luciferase reporter assay system with compounds treated. As baloxavir is known to inhibit IAV polymerase activity, it was selected as a positive control. The pHW2000 plasmids encoding PB2, PB1, PA, and NP proteins, and the pHH21 plasmids expressing negative vRNA-like firefly luciferase (Fluc) RNA were co-transfected into 293T cells, with pRL-TK plasmids expressing renilla luciferase as an internal reference. Luciferase activity was measured 24 h after transfection. Combined with viral replication kinetics experiments and cellular activity assays, the results showed that Emodin, and Andrographolide had no significant effect on IAV polymerase activity (**Figure 3E**). At low concentrations (below 6.25 μM), neither Aloe emodin nor Cryptotanshinone had any effect on polymerase activity. However, at high concentrations (above 12.5 μM), Aloe emodin exhibited inhibitory capacity, while Cryptotanshinone, conversely, demonstrated promoting capacity (**Figure 3E**). To further confirm whether these two compounds directly affect luciferase expression, rather than through the IAV polymerase, additional validation experiments were conducted. 293T cells were transfected with pcDNA3.1-Fluc and pRL-TK and simultaneously treated with different concentrations of Aloe emodin or Cryptotanshinone. The experimental results demonstrated that both compound groups exhibited elevated fluc expression levels at high concentrations (**Figure 3F**). These findings collectively suggest that Aloe emodin may exert inhibitory effects on the IAV polymerase itself. However, the mechanism of action of Cryptotanshinone appears to be independent of IAV polymerase and may instead function through nonspecific regulatory mechanisms, further revealing its potential targets within the host transcription-translational regulatory network—for instance, by enhancing host Pol II-mediated transcriptional activity, modulating the efficiency of host translation mechanisms, and regulating cellular states and stress responses. The absence of such IAV polymerase inhibitory effects in the other two compounds indicates that their antiviral effects may originate from alternative host signaling pathways. These findings provide critical insights for elucidating the underlying mechanisms and guiding subsequent application development.

### 3.4 Four compounds regulate antiviral signaling pathways in host cells

**Figure 4:**
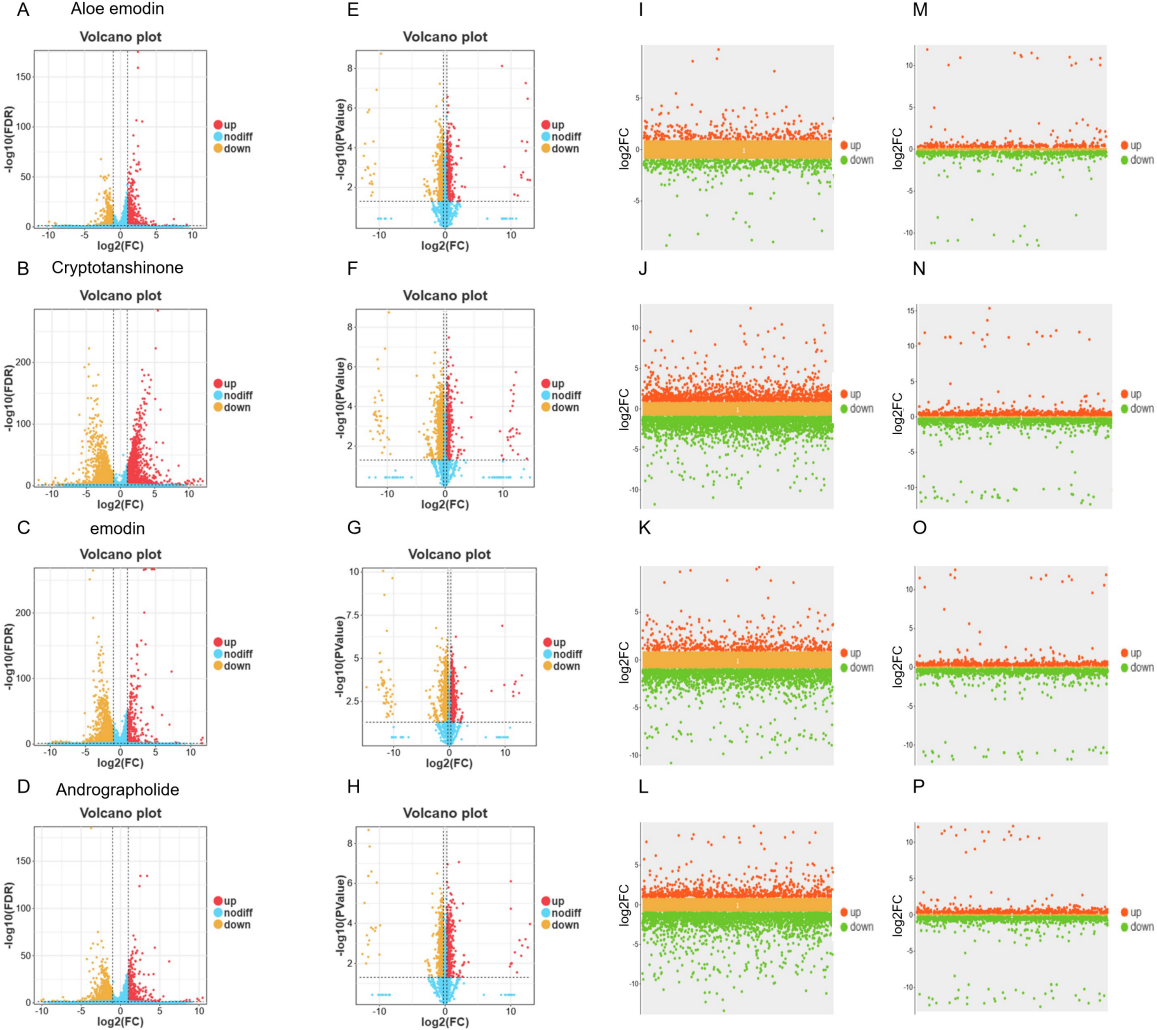
Differential expression analysis of transcriptome and proteome of four compounds in influenza virus-infected and uninfected A549 cells. **(A–D)** Volcano plots of differential expression of the transcriptome in A549 cells not infected with influenza virus after treatment with aloe emodin, cryptosanethone, emodin, and andrographolide; **(E–H)** Volcano plots of differential expression of the proteome in A549 cells not infected with influenza virus after treatment with the aforementioned four compounds; **(I–L)** Scatter plots of differential expression in the transcriptome of A549 cells infected with influenza virus after treatment with the aforementioned four compounds; **(M–P)** Scatter plots of differential expression in the proteome of A549 cells infected with influenza virus after treatment with the aforementioned four compounds.

**Figure 5:**
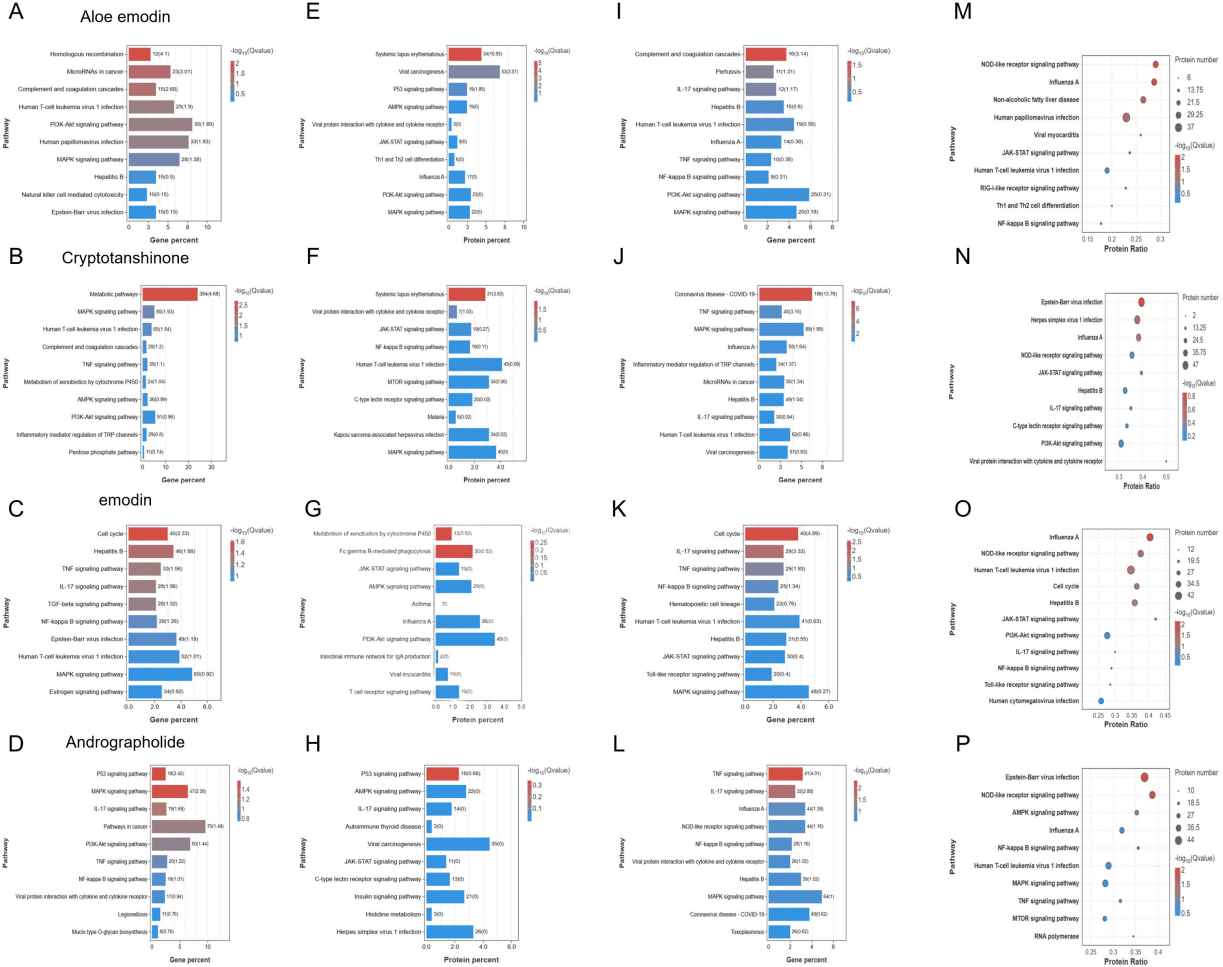
The differential expression of the four compounds in the transcriptome and proteome of A549 cells infected and uninfected with influenza virus were analyzed by pathway enrichment analysis. **(A–D)** Bar plots of transcriptomic pathway enrichment for uninfected A549 cells treated with Aloe emodin, Cryptotanshinone, Emodin, and Andrographolide, showing gene proportions and significance (-log10 (P-value)); **(E–H)** Bar plots of proteomic pathway enrichment for uninfected A549 cells treated with the aforementioned four compounds, displaying protein proportions and significance (-log10 (P-value)); **(I–L)** Bar plots of transcriptomic pathway enrichment for A549 cells infected with influenza virus treated with the aforementioned four compounds; **(M–P)** Bubble plots of proteomic pathway enrichment for A549 cells infected with influenza virus treated with the aforementioned four compounds, with the horizontal axis representing protein enrichment fold, the vertical axis representing pathway names, bubble size indicating protein quantity, and color representing significance (-log10 (P-value)).

To elucidate the antiviral mechanisms of these four compounds against IAV, we conducted transcriptomic and proteomic sequencing analyses to detect cellular changes after compound treatment. A549 cells were either treated with DMSO as a mock or exposed to the four compounds for 24 h, followed by extraction of total RNA and total protein for subsequent transcriptomic and proteomic sequencing. The omics analysis results demonstrated that all four compounds significantly influenced the expression of virus infection-related genes in A549 cells. Compared to the control group, volcano plots (**Figures 4A–D, E–H**) revealed that each compound-treated group exhibited a significant number of differentially expressed genes and proteins. Notably, the most pronounced transcriptional changes were induced by Cryptotanshinone (**Figure 4B**) and Emodin (**Figure 4C**), with the number and distribution range of red (significantly upregulated) and blue (significantly downregulated) dots in the volcano plots exceeding those of Aloe emodin and Andrographolide. Transcriptomic analysis revealed that all compounds collectively enriched pathways including cancer, viral infections, and immune system (**Supplementary Figure 1**). The effects of Aloe emodin were associated with the PI3K-Akt signaling pathway and natural killer cell-mediated cytotoxicity (**Figure 5A**; **Supplementary Figure 1A**). Cryptotanshinone involved a broader network, including TNF, MAPK/PI3K-Akt signaling pathway, and complement cascade (**Figure 5B**; **Supplementary Figure 1B**). The effects of Emodin were highly concentrated in the TNF, NF-κB, and MAPK inflammatory pathways (**Figure 5C**; **Supplementary Figure 1C**). Andrographolide was found to exert multi-target regulation via the TNF, NF-κB, MAPK, and PI3K-Akt signaling pathways (**Figure 5D**; **Supplementary Figure 1D**). Proteomic results validated and expanded these findings. Aloe emodin and Emodin were associated with IAV-related processes and the PI3K-Akt/MAPK signaling pathway (**Figures 5E, G**; **Supplementary Figures 1E, G**). Cryptotanshinone exhibited close association with the NF-κB/MAPK pathway and viral protein-cytokine interactions (**Figure 5F**; **Supplementary Figure 1F**). Andrographolide demonstrated a unique regulatory pattern, likely mediated through C-type lectin receptors and the JAK-STAT signaling pathway (**Figure 5H**; **Supplementary Figure 1H**). In summary, these four compounds can reshape cellular antiviral states by modulating the expression of innate immune and inflammatory response-related genes and proteins in host cells, thereby laying the foundation for subsequent resistance to IAV infection. Among them, activating host innate immunity represents their common antiviral strategy, while each compound exhibits distinct regulatory preferences.

### 3.5 Multi-omics profiling reveals distinct antiviral mechanisms in IAV-infected host cells

Comprehensive multi-omics analysis of IAV-infected A549 cells treated with four compounds revealed specific roles of these compounds in reprogramming the host’s response to viral attack. Unlike the basal regulatory effects observed in uninfected cells, this analysis captured dynamic, infection-specific remodeling of cellular pathways by each compound. Transcriptomic analysis of infected cells demonstrated that Aloe emodin, Cryptotanshinone, Emodin, and Andrographolide induced significant and widespread changes in host gene expression post-infection (**Figures 4I–L**). Pathway enrichment analysis indicated that all compounds significantly regulated core antiviral defense gene networks, including pathways annotated as viral infectious diseases, immune system, and signal transduction (**Supplementary Figure 2**). Notably, each compound exhibited unique regulatory characteristics: Aloe emodin primarily affected IAV, TNF/NF-κB, and PI3K-Akt/MAPK pathways (**Figure 5I**; **Supplementary Figure 2A**); Cryptotanshinone was significantly enriched in IAV, TNF, and MAPK signaling cascades (**Figure 5J**; **Supplementary Figure 2B**); Emodin was associated with TNF, NF-κB, MAPK, and Toll-like receptor pathways (**Figure 5K**; **Supplementary Figure 2C**); and Andrographolide influenced IAV pathways, viral protein-cytokine receptor interactions, and the integrated TNF/NF-κB/MAPK axis (**Figure 5L**; **Supplementary Figure 2D**). Proteomic analysis of identical infection-treatment scenarios confirmed and expanded these findings, revealing significant changes in protein abundance across key functional categories (**Figures 4M–P**). The data further highlighted compound-specific regulation of critical infection response pathways. Aloe emodin and Emodin were associated with the modulation of NOD-like receptor signaling and the JAK-STAT/NF-κB pathway (**Figures 5M, O**, **Supplementary Figures 2E, G**). Cryptotanshinone was specifically linked to viral protein-cytokine receptor interactions, in addition to its involvement in NOD-like receptor signaling (**Figure 5N**; **Supplementary Figure 2F**). Andrographolide demonstrated its participation in both NOD-like receptor signaling and the TNF/NF-κB/MAPK network (**Figure 5P**; **Supplementary Figure 2H**). Collectively, multi-omics analysis of infected cells indicated that these four anti-IAV compounds not only regulate static host pathways but also actively and differentially reconstruct the host’s transcriptional and translational landscapes in response to IAV infection. Each compound targets distinct subpopulations of core antiviral and immunomodulatory pathways, highlighting their unique host-directed mechanisms in pathophysiological contexts.

## 4 Discussion

In this study, a multi-tiered phenotypic screening strategy was designed and executed to systematically identify natural compounds with anti-IAV activity from a library enriched with molecules derived from traditional medicine systems. This approach, which integrated an initial knowledge-based curation with a high-throughput GFP-reporter virus assay and subsequent validation through viral replication kinetics, proved highly effective. It enabled us to discover four structurally distinct compounds, Aloe emodin, Cryptotanshinone, Emodin, and Andrographolide, that consistently and significantly suppressed IAV (H1N1) replication in both MDCK and human lung epithelial A549 cell lines. Crucially, beyond their confirmed antiviral efficacy, preliminary mechanistic investigations indicated that these compounds operate through predominantly host-centric pathways. This finding underscores their substantial potential as novel chemical scaffolds for the development of HDTs, a strategic paradigm aimed at targeting stable host factors co-opted by the virus, thereby offering a promising avenue to circumvent the persistent challenge of viral drug resistance.

The strategy of HDTs represents a paradigm shift in antiviral drug development, driven by the need to overcome the rapid emergence of resistance associated with direct-acting antivirals (Chitalia and Munawar, 2020; Thom and D’Elia, 2024). By targeting evolutionarily stable host factors that are essential for viral replication, HDTs offer the potential for broad-spectrum activity and a higher genetic barrier to resistance (Thom and D’Elia, 2024). This approach has gained significant momentum beyond academia, with initiatives such as the U.S. BARDA’s Host-Directed Therapeutics program actively advancing candidates into clinical trials for diseases like COVID-19, underscoring its translational potential (Beigel et al., 2019; Kaufmann et al., 2018; Wu et al., 2022). Several drugs with host-directed mechanisms are already established in clinical practice for other viral infections. A prime example is maraviroc, a CCR5 antagonist approved in 2007 for HIV treatment, which works by blocking the viral co-receptor on host cells to prevent entry (Lieberman-Blum et al., 2008). These successes validate the HDT paradigm and provide a roadmap for its application against other pathogens. However, the landscape for IAV-specific HDTs is markedly different. Although our understanding of host factors in IAV replication is increasing and research is promising (Li et al., 2025; Maes et al., 2024; Yang et al., 2022), no HDTs have yet been approved for clinical use against IAV. Currently, first-line treatments for IAV remain limited to direct-acting antiviral drugs, such as neuraminidase inhibitors (e.g., oseltamivir) (Doshi et al., 2016) and RNA polymerase inhibitors (e.g., baloxavir marboxil and favipiravir) (Dufrasne, 2021; Shiraki and Daikoku, 2020). This situation highlights a significant unmet need and valuable opportunities for innovative interventions.

Our multi-tiered screening approach has thus led to the identification of four natural compounds whose significance was underscored by the pressing need for novel antiviral strategies. The transcriptomic analysis of compound-treated, IAV-infected A549 cells revealed that these four compounds modulate distinct yet complementary host antiviral signaling networks. These network modulations highlight their potential as multi-target host-directed agents. Specifically, Aloe emodin significantly influenced pathways related to PI3K-Akt signaling (Zhao et al., 2023) and natural killer cell-mediated cytotoxicity (Morson et al., 2025), which are known to regulate viral entry and innate immune surveillance. Cryptotanshinone exhibited broad modulation of TNF and MAPK/PI3K-Akt cascades, along with complement activation, suggesting a role in tempering excessive inflammation while maintaining antiviral defense (Xie et al., 2021). Emodin strongly targeted TNF, NF-κB, and MAPK pathways, which are central to cytokine production and apoptosis during IAV infection, indicating a potential for mitigating immunopathology, also consistent with previous findings (Dai et al., 2017). Andrographolide displayed a multi-faceted regulatory signature involving TNF, NF-κB, MAPK, and PI3K-Akt pathways, with additional engagement of C-type lectin receptor and JAK-STAT signaling—a profile suggestive of enhanced interferon-mediated antiviral response and immune modulation (Geng et al., 2021). Importantly, proteomic data corroborated these transcriptomic patterns, confirming that these compounds induce coherent changes at both the mRNA and protein levels in key infection-responsive pathways such as viral protein–cytokine receptor interactions, NOD-like receptor signaling, and integrated NF-κB/MAPK networks. This concordance across omics layers strengthens the conclusion that each compound rewires the host cellular environment through distinct but complementary mechanisms, collectively disrupting the virus–host interface essential for IAV replication. These findings align with the paradigm of HDTs, as the compounds appear to bolster host defense mechanisms or disrupt host dependencies of the virus, rather than directly inhibiting viral enzymes, the conclusion supported by the polymerase activity assays showing limited direct inhibition compared with baloxavir group. However, the multi-target nature of these natural chemical compounds, evidenced by their engagement with different host pathways, may also be a double-edged sword. While it may enhance efficacy and reduce the likelihood of resistance by applying multi-faceted pressure on the viral life cycle, it also raises the possibility of off-target effects, as reflected in the cell type-dependent cytotoxicity we observed. For instance, a systematic chemogenomic study demonstrated that small-molecule drugs commonly exhibit off-target effects, and their biological impacts (including cytotoxicity) were strongly dependent on the genotypic context of the cell line, directly illustrating the differential safety risks that multi-target compounds might pose across different cellular backgrounds (Garnett et al., 2012). In fact, off-target effects are a leading cause of candidate drug attrition in late-stage development due to safety concerns (Cao et al., 2024; Gould and Templin, 2023). Future research should focus on elucidating the precise molecular targets within these pathways and optimizing compound structures to improve selectivity and therapeutic index.

Nevertheless, several limitations of our study warrant consideration and guide future directions. First, while our transcriptomic data implicate modulation of host pathways (e.g., RIG-I and NF-κB signaling), the precise molecular targets and the exact mode of action for each compound remain to be elucidated. Future studies employing chemical proteomics or affinity-based pull-down assays are essential to identify the direct protein interactors, a critical step for rational drug optimization and understanding potential off-target effects (Edwards and Hsu, 2025; Wiest and Kielkowski, 2024). Second, the cell-type-dependent cytotoxicity observed, underscores the critical need for rigorous *in vivo* safety and pharmacokinetic profiling prior to any clinical translation. The translational potential of any HDT candidate must be evaluated in physiologically relevant animal models, as is standard practice in advancing natural chemical compound-derived anti-IAV agents toward clinical application. Third, although our dual-luciferase assay suggested that Aloe emodin might influence viral polymerase activity at high concentrations, its primary mechanism at physiologically relevant doses appears to be host-mediated. Future work should explore potential synergistic effects when these host-directed compounds are combined with existing direct-acting antivirals. This strategy of combining agents with distinct mechanisms, which target both the host and the virus, has shown great promise in creating more robust and resistance-resistant therapeutic regimens, as evidenced by recent advances in the treatment of other viral infections such as hepatitis (Deng et al., 2023; Nasser et al., 2023). The exploration of such combinations represents a logical and promising next step for developing these multi-target natural compounds into effective anti-IAV therapies.

In conclusion, this study identifies four natural compounds with potent anti-influenza activity, and through integrated phenotypic and transcriptomic analyses, provides preliminary evidence that their efficacy stems from distinct host-directed mechanisms. These positions them as novel chemical scaffolds that address the critical gap in current therapies by targeting host pathways, a strategy with the potential for broader efficacy and a higher barrier to resistance. To advance these leads, future work must prioritize *in vivo* efficacy and safety validation, precise deconvolution of molecular targets using genetic and proteomic tools, and the exploration of synergistic combinations with existing antivirals, ultimately paving the way for durable therapeutic strategies against influenza evolution.

## Data Availability

The transcriptomics and proteomics data have been uploaded to the National Microbial Science Data Center (https://nmdc.cn/) with submission number SUB1771655082228.

## Supplementary material

Supplementary Figure 1 Four compounds regulate antiviral signaling pathways in host cells. The pathways co-enriched by the four compounds all involve cancer, viral infectious diseases, and the immune system. **(A-D)** Transcriptomic enrichment pathways of the four compounds; **(E-H)** Proteomic enrichment pathways of the four compounds.

Supplementary Figure 2 These four compounds exhibit distinct antiviral mechanisms against IAV-infected host cells. All four compounds significantly modulated the gene networks at the core of antiviral defense, including those annotated for viral infectious diseases, immune system, and signal transduction. **(A-D)** Transcriptomic enrichment pathways of the four compounds; **(E-H)** Proteomic enrichment pathways of the four compounds.
