# Supplementary figures and images for: Integrating prior knowledge inference with computational multi-omics analysis to reveal host antiviral networks of natural compounds against influenza A virus
Source: Front Cell Infect Microbiol. 2026 Mar 2;16:1771638. doi: 10.3389/fcimb.2026.1771638 (PMC12993371; doi:10.3389/fcimb.2026.1771638)

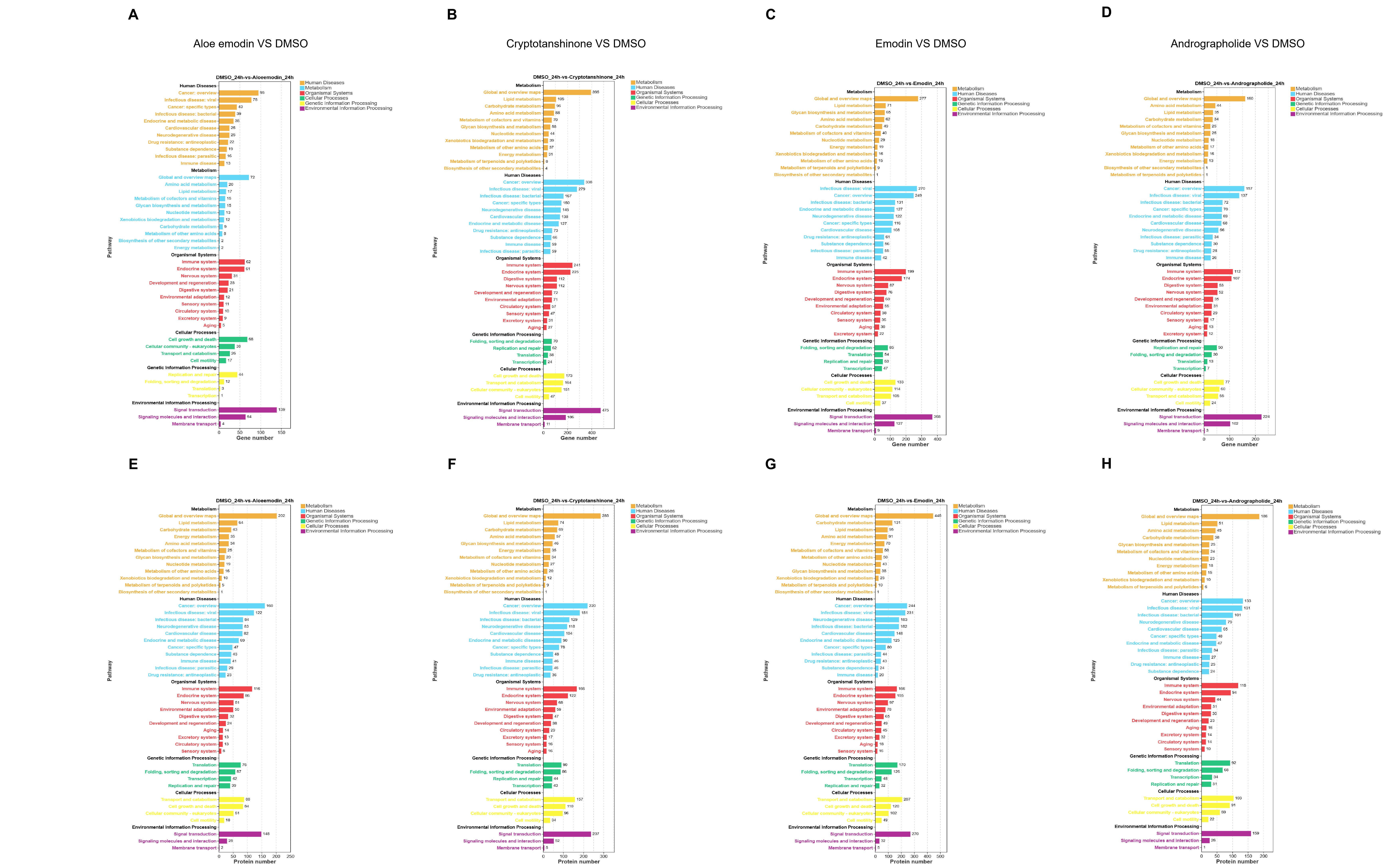

Supplement: Supplementary Figure 1 — Four compounds regulate antiviral signaling pathways in host cells. The pathways co-enriched by the four compounds all involve cancer, viral infectious diseases, and the immune system. (A-D) Transcriptomic enrichment pathways of the four compounds; (E-H) Proteomic enrichment pathways of the four compounds. [file Image1.jpeg]

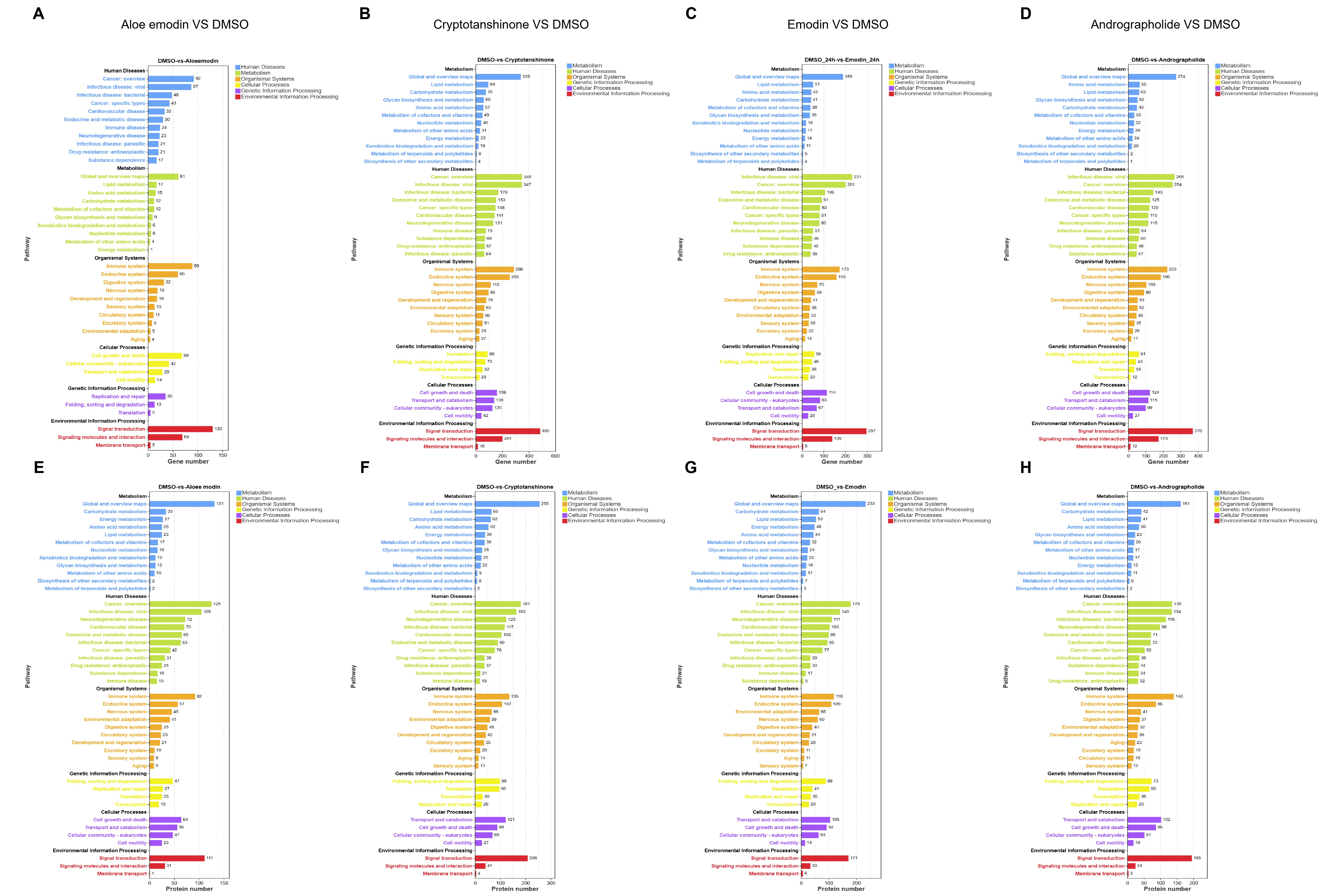

Supplement: Supplementary Figure 2 — These four compounds exhibit distinct antiviral mechanisms against IAV-infected host cells. All four compounds significantly modulated the gene networks at the core of antiviral defense, including those annotated for viral infectious diseases, immune system, and signal transduction. (A-D) Transcriptomic enrichment pathways of the four compounds; (E-H) Proteomic enrichment pathways of the four compounds. [file Image2.jpeg]
