# Supplementary material for: Integrating prior knowledge inference with computational multi-omics analysis to reveal host antiviral networks of natural compounds against influenza A virus
Source: Front Cell Infect Microbiol. 2026 Mar 2;16:1771638. doi: 10.3389/fcimb.2026.1771638 (PMC12993371; doi:10.3389/fcimb.2026.1771638)
Supplement: Supplementary file 3 [file Table1.docx]

**Supplementary table 1 Basic information of 20 compounds**

| **Name** | **molecular weight** | **Formula** | **pigment** |
| --- | --- | --- | --- |
| Forsythiaside A | 624.59 | C_29_H_36_O_15_ | Off-white to yellow |
| Coptisine Sulfate | 417.39 | C_19_H_15_NO_8_S | Yellow to orange |
| Costunolide | 232.32 | C_15_H_20_O_2_ | White to yellow |
| 14-Deoxyandrographolide | 334.45 | C_20_H_30_O_4_ | White to off-white |
| Farrerol | 300.31 | C_17_H_16_O_5_ | White to light yellow |
| Andrographolide | 350.45 | C_20_H_30_O_5_ | White to off-white |
| Emodin | 270.24 | C_15_H_10_O_5_ | Yellow to orange |
| Cryptotanshinone | 296.36 | C_19_H_20_O_3_ | Pink to red |
| Ginsenoside Rg1 | 801.01 | C_42_H_72_O_14_ | White to off-white |
| Berberine chloride | 371.81 | C_20_H_18_ClNO_4_ | Light yellow to yellow |
| Aloe emodin | 270.24 | C_15_H_10_O_5_ | Brown to orange |
| 18α-Glycyrrhetinic acid | 470.68 | C_30_H_46_O_4_ | White to off-white |
| Puerarin | 416.38 | C_21_H_20_O_9_ | White to off-white |
| 5-O-Methylvisammioside | 452.45 | C_22_H_28_O_10_ | White to off-white |
| Stachydrine hydrochloride | 179.64 | C_7_H_14_ClNO_2_ | White to off-white |
| Ferulic acid | 194.18 | C_10_H_10_O_4_ | Off-white to yellow |
| Rosmarinic acid | 360.31 | C_18_H_16_O_8_ | Off-white to light brown |
| Leonurine hydrochloride | 347.79 | C_14_H_22_ClN_3_O_5_ | White to off-white |
| Paeoniflorin | 480.46 | C_23_H_28_O_11_ | White to off-white |
| Liquiritin | 418.39 | C_21_H_22_O_9_ | White to off-white |
